# Widespread Community Transmission of Hepatitis A Virus Following an Outbreak at a Local Restaurant — Virginia, September 2021–September 2022

**DOI:** 10.15585/mmwr.mm7214a2

**Published:** 2023-04-07

**Authors:** Meagan J. Helmick, Cynthia B. Morrow, J. Hope White, Paige Bordwine

**Affiliations:** ^1^Division of Surveillance and Investigation, Office of Epidemiology, Virginia Department of Health; ^2^Roanoke City and Alleghany Health Districts, Virginia Department of Health.

Hepatitis A is a vaccine-preventable liver infection caused by the hepatitis A virus (HAV); it is transmitted through ingestion of food or drink that has been contaminated by small amounts of infected stool, or through direct contact, including sexual contact, with a person who is infected (*1*). After years of historically low rates of hepatitis A in the United States, the incidence began increasing in 2016, with outbreaks characterized by person-to-person HAV transmission among persons who use drugs, persons experiencing homelessness, and men who have sex with men (*2**,**3*). As of September 2022, 13 states were experiencing outbreaks, including Virginia (*3*). In September 2021, the Roanoke City and Alleghany Health Districts (RCAHD) in southwestern Virginia investigated an outbreak of hepatitis A. The outbreak, which resulted in 51 cases, 31 hospitalizations, and three deaths, was associated with a food handler who was infected. After the outbreak, the community experienced ongoing person-to-person transmission of HAV, predominantly among persons who use injection drugs. As of September 30, 2022,* an additional 98 cases had been reported to RCAHD. The initial outbreak and community transmission have exceeded US$3 million in estimated direct costs (*4**,**5*). This report describes the initial outbreak and the ongoing community transmission of HAV. Increasing vaccination coverage among persons with risk factors for hepatitis A infection is important, including among those who use drugs. Strengthening community partnerships between public health officials and organizations that employ persons with risk factors for acquisition of HAV could help to prevent infections and outbreaks.

## Initial Outbreak Investigation (September–November 2021)

RCAHD serves a population of approximately 280,000 persons. The districts have previously experienced a low hepatitis A incidence, with only six cases reported during January 1, 2019–December 31, 2020, and none through August 2021. In mid-September 2021, however, five hepatitis A cases were reported during a single week. Initial case investigations identified a local restaurant chain as a common source of exposure. Further investigation identified the index patient as an unvaccinated food handler who had risk factors for hepatitis A and who had worked at three locations of the same restaurant chain; however, this person delayed seeking medical attention for more than 2 weeks after symptom onset and did not disclose being employed as a food handler at that time. Based on the index patient’s symptom onset date and last day worked, the exposure period for patrons who ate at any of the three restaurants was determined to be August 10–26, 2021. When the outbreak investigation was closed on November 20, 2021, 51 restaurant-associated cases had been identified (Figure).^†^ The median patient age was 64 years (range = 30–86 years) (Table).

**FIGURE:**
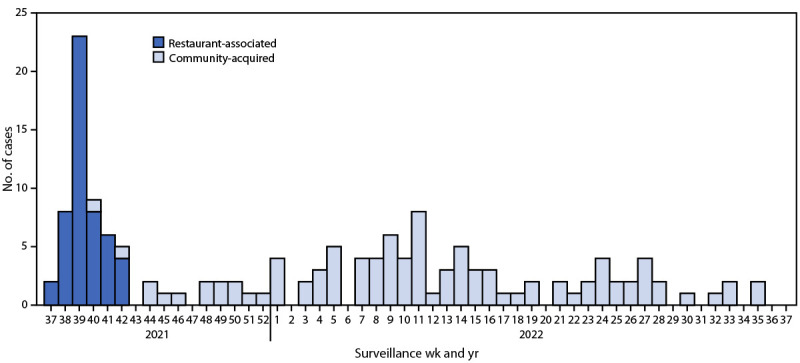
Confirmed hepatitis A cases (N = 149), by surveillance week of diagnosis and outbreak classification*— Virginia, September 2021–September 2022 Abbreviation: CSTE = Council of State and Territorial Epidemiologists. * A restaurant-associated case of hepatitis A was defined as an illness meeting the Council of State and Territorial Epidemiologists (CSTE) confirmed case criteria (https://ndc.services.cdc.gov/case-definitions/hepatitis-a-acute-2019/) in a person who dined at any of the three restaurant locations during August 10–26, 2021, or who had close contact with the index patient. A community-acquired case of hepatitis A was defined as an illness meeting the CSTE confirmed case criteria of hepatitis A in a person who did not dine at any of the restaurants during the exposure period, and had no contact with a patient who did dine at any of the restaurants during September 2021–September 2022.

**TABLE:** Characteristics of restaurant-associated and community-acquired hepatitis A cases* — Virginia, 2021–2022

Characteristic	No. (%) of cases	
	Restaurant-associated (n = 51)	Community-acquired (n = 98)
Demographic/Comorbidity		
Median age, yrs (range)	64 (30–86)	40 (24–89)
Male	29 (56.9)	55 (56.1)
Non-Hispanic	51 (100.0)	96 (98.0)
White	50 (98.0)	88 (90.0)
Reported diabetes	11 (21.6)	4 (4.1)
**Signs and symptoms**		
Abdominal pain	22 (43.1)	59 (60.2)
Anorexia	28 (54.9)	18 (18.4)
Arthritis	3 (5.9)	0 (—)
Chills	11 (21.6)	15 (15.3)
Dark-colored urine	36 (70.6)	33 (33.7)
Diarrhea	15 (29.4)	11 (11.2)
Fatigue or malaise	44 (86.3)	37 (37.8)
Fever	26 (51.0)	17 (17.4)
Itching	5 (10.0)	3 (3.1)
Jaundice	27 (52.9)	53 (54.1)
Light or clay stools	14 (27.5)	12 (12.2)
Muscle aches	14 (27.5)	10 (10.2)
Nausea	38 (74.5)	54 (55.1)
Sweats	5 (10.0)	1 (1.0)
Vomiting	20 (39.2)	38 (38.8)
Weight loss	6 (11.8)	3 (3.1)
**Outcomes**		
Hospitalized	31 (60.8)	64 (65.3)
Died from illness	3 (5.9)	0 (—)
Received liver transplant	1 (2.0)	0 (—)
**Risk factors**		
Homelessness	0 (—)	12 (12.2)
Any drug use	2 (3.9)	84 (85.7)
Injection drug use	0 (—)	73 (74.5)
Noninjection drug use	2 (3.9)	69 (70.4)
Male-to-male sexual contact	2 (3.9)	1 (1.0)
Incarceration	0 (—)	8 (8.2)

* Missing data were treated as “no” for the purpose of analysis.

## Sustained Community Transmission (Ongoing)

In October 2021, RCAHD began to receive reports of hepatitis A cases that were not directly associated with the index patient or restaurant outbreak. By the end of 2021, 13 additional cases were reported to RCAHD. Sustained community transmission continued into 2022, with 98 total cases reported through September 2022, including 64 hospitalizations (Figure). An identical genotype IB sequence was identified in eight of the nine specimens submitted from the restaurant outbreak and from all five sequenced community case specimens.

Although a clear temporal association between the restaurant outbreak and two of the community cases was demonstrated, limited contact information for most of the persons with community-acquired cases hampered the ability to conclusively identify an epidemiologic link between the restaurant outbreak and sustained person-to-person community transmission. Among the 98 cases, only 40 (40.8%) patients were interviewed by public health officials, either because of insufficient contact information (57) or refusal to be interviewed (one). Public health officials conducted a comprehensive medical record review of all cases to identify patient risk factors, contacts, and exposures. Among persons with community-acquired cases, the most commonly identified hepatitis A risk factors were any drug use (85.7%) (including injection drug use [74.5%]) and experiencing homelessness (12.2%) (Table).

## Public Health Response

When the restaurant chain was identified as a common risk during the initial outbreak, RCAHD’s environmental health specialists performed risk assessments at each site and determined that the index patient had ungloved-hand contact with ready-to-eat foods while infectious. RCAHD personnel worked with the restaurant’s management and the state health department to coordinate risk communication.^§^ The first press release was issued on September 24, 2021, recommending that persons with symptoms consistent with hepatitis A seek medical care. Because restaurant exposure was identified outside the 2-week period during which postexposure prophylaxis (PEP) with hepatitis A vaccine or immune globulin is recommended, PEP was not advised for the public. Although RCAHD encouraged and facilitated hepatitis A vaccination for employees of the affected restaurants and close contacts of patients, including providing on-site vaccinations, fewer than 20% of eligible employees were vaccinated.

Similar public health responses were implemented in 2022 when three hepatitis A cases were diagnosed in local restaurant workers. Throughout 2022, RCAHD partnered with community-based organizations to increase education about hepatitis A and access to the hepatitis A vaccine for persons at risk for acquiring HAV. By September 30, 2022, >2,500 hepatitis A vaccine doses had been administered through these efforts.

The financial toll of these outbreaks has exceeded US$3 million in estimated direct costs for hospitalizations ($16,232 per hospitalization), one liver transplant ($1,427,805), and hepatitis A vaccines^¶^ (*4**,**5*). This estimate does not include indirect costs such as personnel costs or missed wages for patients (*4**,**5*).

## Discussion

In hepatitis A outbreaks, rapid source identification is critical to interrupting transmission. In this outbreak, RCAHD quickly identified the source; however, because of the long incubation period (15–50 days), a delay in the index patient’s seeking medical attention, and that person not disclosing their occupation during the initial interview, widespread transmission, resulting in 51 cases, 31 hospitalizations, and three deaths, had already occurred. The severity of outcomes associated with the restaurant-associated outbreak was likely due to a combination of the high median patient age and prevalence of comorbidities.

The unvaccinated index patient in this outbreak had risk factors for which hepatitis A vaccine is routinely recommended. Hepatitis A vaccines are safe and effective and became part of the routine childhood vaccination schedule in 2006 (*6*). Current adult vaccination recommendations focus on “any person who requests vaccination” (*7*) and disproportionately affected populations that include persons who use drugs, men who have sex with men, persons experiencing homelessness, and international travelers. In the United States during 2011–2016, approximately two thirds of persons who reported injection drug use (67%) and men who have sex with men (64%) reported that they had not been vaccinated against hepatitis A (*8*).

Despite the association of drug use with widespread U.S. hepatitis A outbreaks, vaccination efforts targeting persons who use drugs is challenging for many reasons, including behavioral health issues, limited engagement in health care systems, and transportation problems (*9*). A 2013 Substance Abuse and Mental Health Services Administration study found that, among industries, the restaurant industry had the highest rates of drug use, with nearly 20% of food service workers reporting drug use during the preceding month (*10*). In 2019, the U.S. food industry employed 15.3 million persons, suggesting that nearly 3 million food industry workers might be using drugs.** To improve hepatitis A vaccination coverage among persons who use drugs, public health agencies can explore partnerships with businesses that might employ persons at higher risk for hepatitis A. Persons who use drugs might not disclose being in a high-risk category recommended for hepatitis A vaccination, but might consider vaccination if encouraged by their employer. Health care professionals and public health officials should continue to encourage vaccination among disproportionately affected populations.

The findings in this report are subject to at least four limitations. First, the initial outbreak began during the COVID-19 Delta variant surge, and community transmission of HAV continued during the Omicron variant surge. Pandemic-related response activities in the community might have resulted in underreporting of cases. Second, extensive media coverage of the hepatitis A outbreak might have increased hepatitis A testing in the community, leading to more diagnoses of cases not associated with the outbreak. Third, RCAHD encountered challenges to interviewing patients, which could have resulted in underreporting of risk factors, epidemiologic links, and specific symptoms. This underreporting limited RCAHD’s ability to identify direct epidemiologic linkages between restaurant-associated cases and subsequent community transmission. Finally, because the specific genotype IB sequence identified in these outbreaks is not uncommon, a causal association between the restaurant outbreak and the ongoing person-to-person transmission could not be established.

After years of limited HAV transmission in the Roanoke, Virginia area, hepatitis A cases increased sharply in the community following an initial outbreak associated with an unvaccinated food handler with hepatitis A risk factors. Increasing awareness of risk factors for hepatitis A, particularly among food handlers, and increasing vaccine access for persons at risk, particularly those who use drugs, could help prevent similar outbreaks in other communities.

**Summary**

**What is already known about this topic?**

U.S. hepatitis A incidence has been increasing since 2016.

**What is added by this report?**

In 2021, a hepatitis A outbreak in Virginia traced to an unvaccinated food handler resulted in 51 cases, 31 hospitalizations, and three deaths. As of September 30, 2022, an additional 98 community hepatitis A cases had been reported in the Roanoke City and Alleghany Health Districts.

**What are the implications for public health practice?**

Public health partnerships with businesses and other community partners (e.g., harm reduction programs) might increase hepatitis A vaccination among persons at risk for this infection, while also reducing the stigmatization of hepatitis A-associated risk factors.

## Footnotes

* During October 1, 2022–March 31, 2023, seven additional hepatitis A cases were identified in association with this outbreak.

† A restaurant-associated case of hepatitis A was defined as an illness meeting the Council of State and Territorial Epidemiologists (CSTE) confirmed case criteria (https://ndc.services.cdc.gov/case-definitions/hepatitis-a-acute-2019/) in a person who dined at any of the three restaurant locations during August 10–26, 2021, or who had close contact with the index patient. A community-acquired case of hepatitis A was defined as an illness meeting the CSTE confirmed case criteria of hepatitis A in a person who did not dine at any of the restaurants during the exposure period, and had no contact with a patient who did dine at any of the restaurants during September 2021–September 2022.

§
https://www.vdh.virginia.gov/news/2020-regional-news-releases/health-alert-potential-hepatitis-a-exposure-at-local-restaurant/

¶ https://www.cdc.gov/vaccines/programs/vfc/awardees/vaccine-management/price-list/index.html (Accessed October 17, 2022).

**
https://restaurant.org/getmedia/21a36a65-d5d4-41d0-af5c-737ab545d65a/nra-data-brief-restaurant-employee-demographics-march-2022.pdf
